# High fidelity medical simulation in the difficult environment of a helicopter: feasibility, self-efficacy and cost

**DOI:** 10.1186/1472-6920-6-49

**Published:** 2006-10-05

**Authors:** Stewart W Wright, Christopher J Lindsell, William R Hinckley, Annette Williams, Carolyn Holland, Christopher H Lewis, Gail Heimburger

**Affiliations:** 1 University of Cincinnati, Department of Emergency Medicine, Cincinnati, Ohio, USA; 2 University of Southampton, Institute of Sound and Vibration Research, Human Factors Research Unit, UK; 3 University of Cincinnati College of Medicine, Dean's Office, Cincinnati, Ohio, USA

## Abstract

**Background:**

This study assessed the feasibility, self-efficacy and cost of providing a high fidelity medical simulation experience in the difficult environment of an air ambulance helicopter.

**Methods:**

Seven of 12 EM residents in their first postgraduate year participated in an EMS flight simulation as the flight physician. The simulation used the Laerdal SimMan™ to present a cardiac and a trauma case in an EMS helicopter while running at flight idle. Before and after the simulation, subjects completed visual analog scales and a semi-structured interview to measure their self-efficacy, i.e. comfort with their ability to treat patients in the helicopter, and recognition of obstacles to care in the helicopter environment. After all 12 residents had completed their first non-simulated flight as the flight physician; they were surveyed about self-assessed comfort and perceived value of the simulation. Continuous data were compared between pre- and post-simulation using a paired samples t-test, and between residents participating in the simulation and those who did not using an independent samples t-test. Categorical data were compared using Fisher's exact test. Cost data for the simulation experience were estimated by the investigators.

**Results:**

The simulations functioned correctly 5 out of 7 times; suggesting some refinement is necessary. Cost data indicated a monetary cost of $440 and a time cost of 22 hours of skilled instructor time. The simulation and non-simulation groups were similar in their demographics and pre-hospital experiences. The simulation did not improve residents' self-assessed comfort prior to their first flight (p > 0.234), but did improve understanding of the obstacles to patient care in the helicopter (p = 0.029). Every resident undertaking the simulation agreed it was educational and it should be included in their training. Qualitative data suggested residents would benefit from high fidelity simulation in other environments, including ground transport and for running codes in hospital.

**Conclusion:**

It is feasible to provide a high fidelity medical simulation experience in the difficult environment of the air ambulance helicopter, although further experience is necessary to eliminate practical problems. Simulation improves recognition of the challenges present and provides an important opportunity for training in challenging environments. However, use of simulation technology is expensive both in terms of monetary outlay and of personnel involvement. The benefits of this technology must be weighed against the cost for each institution.

## Background

Emergency medicine training is a complicated endeavor. Emergency medicine residents strive to master a broad knowledge base while attempting to provide quality, efficient care in the world's teaching hospitals. Training programs must provide high quality care while simultaneously allowing novices the opportunity for experience. This apprentice-modeled, experiential learning design has been traditionally summarized by the dictum of "see one, do one, teach one". This model is no longer considered optimal[1,2]. The 1999 Institute of Medicine report on medical errors and patient safety has placed renewed emphasis on training modalities and forced non-direct patient care teaching activities to the forefront. Methods to improve resident critical care skills without placing patients at risk are fundamental for maximizing patient safety while effectively educating residents. High fidelity medical simulation training presents such a method[3].

Medical simulation is becoming a mainstream method of medical education [4-8] and evaluation[9,10]. Medical simulators have been used to teach professionalism, [11] teamwork,[12,13] cognitive strategies,[14] crisis resource management,[15] and a crisis resource management model curriculum for undergraduates utilizing simulators has been proposed[16]. Users of the modality have reported high student satisfaction[17,18].

Medical simulation has mainly used static displays of mannequins with multiple operators and teams of learners in simulated patient rooms. Little high fidelity medical simulation has been attempted in the prehospital setting where providers must act alone under far from ideal conditions, where environmental noise, vibration and motion all act to reduce performance. The air medical setting is particularly challenging. High levels of noise and vibration in helicopters are associated with the revolution rates of the rotors, gearbox, engine and other rotating parts. The high noise and vibration result in additional stresses and difficulties for the patient, medical equipment, and crew. Cabin noise levels of around 97 dB(A) are typical in air medical helicopters during cruise,[19] and may be greater during take-off and landing. These noise levels can cause major difficulties in speech communication, and permanent hearing loss in frequently exposed personnel. The noise levels make it necessary to use amplified intercom systems with noise attenuating headsets to provide adequate speech intelligibility and hearing protection for the crew. Badly fitting intercom headsets caused by, for instance, wearing glasses, can contribute to speech intelligibility problems for medical personnel[20] The inability to hear breath sounds, even when using an amplified stethoscope, has also been reported[21] Other problems that have been reported due to noise in air medical helicopters include the difficulties in hearing auditory signals and alarms from medical equipment,[22] making it necessary to rely on scanning of visual displays to detect alarm conditions, and resulting in the possibility of prolonged periods before alarm detection. Helicopter vibration transmitted to a visual display can also result in the blurring of small detail, exacerbating problems caused by the need to relay on visual information[23]. Although the combined noise and vibration environment typical of a helicopter does not affect cognitive performance (short term memory and reaction time),[24] and direct interference with tasks involving fine manipulation is unlikely at the typical frequencies of vibration in air medical helicopters,[23] the combined noise and vibration is rated as more annoying and more difficult than other conditions[24]. There is a need for a portable high fidelity simulator that can be used for training individuals in this adverse environment. The Laerdal SimMan™ is one such simulator and may provide trainees with the experience necessary to cope with the difficulties of the environment.

The emergency medicine residency at our institution is a four-year training program that includes extensive experience as a member of a helicopter emergency medical services flight crew consisting of a flight nurse and a flight physician. At the beginning of the second post-graduate year, physicians fly on a BK-117 EMS helicopter as the primary flight physician. Current air medical training consists of a rigorous two-day didactic course, and a system of "buddy flights". These flights allow residents at the end of their first year to experience the flight medicine environment under the supervision of a more experienced resident as the primary flight physician.

We proposed that the current training can be significantly augmented by a novel simulation experience utilizing the Laerdal SimMan™. We hypothesized that a high fidelity simulated flight medicine experience can be given to trainees in the difficult environment of a modern air ambulance helicopter. Secondarily, we used qualitative and quantitative means to attempt to measure self-efficacy, i.e. what the residents felt they gained, through this simulation. Similarly we estimate the manpower and monetary cost of the project

## Methods

This was a pre- post-educational intervention study approved by the Institutional Review Board. Both quantitative and qualitative data were gathered to evaluate feasibility and perceived value of the simulation.

## Subjects

Twelve emergency medicine residents completing their first year of training were invited to participate in the training. Invitation was by electronic mail and verbal communication by the primary investigator during weekly educational conferences. All twelve residents were invited to participate in the educational experience of the simulation irrespective of their consent to be enrolled in the study group. Seven residents were available for the educational experience and all seven elected to participate in the trial; this group was designated the simulation group. The five residents who were unavailable did not participate because of clinical and scheduling conflicts; this group was designated the non-simulation group and received no experience with the medical simulator. All twelve residents participated in the standard "buddy flight" system of training.

### Educational intervention

**Figure 1:**
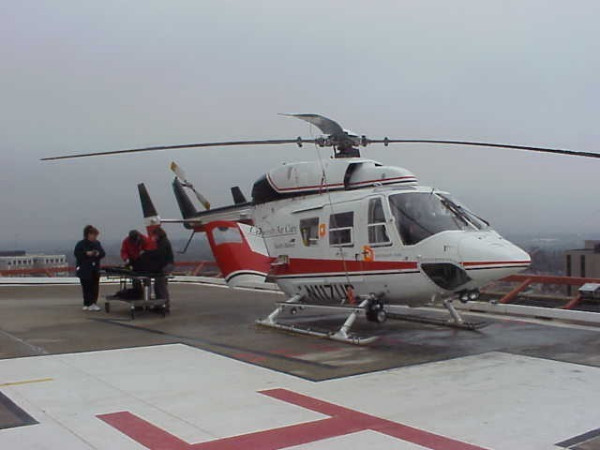
BK-117 EMS Air Ambulance Prepared for Flight.

**Figure 2:**
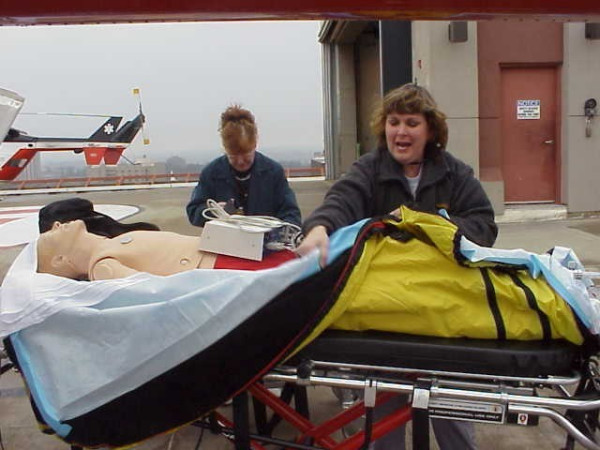
Simulator Ready for Loading into Position.

**Figure 3:**
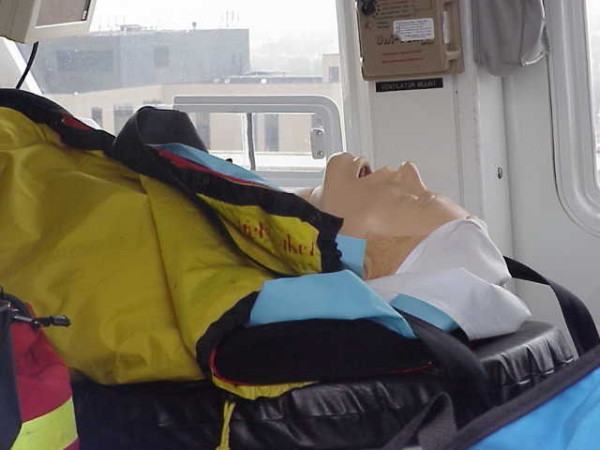
Simulator in Position.

**Figure 4:**
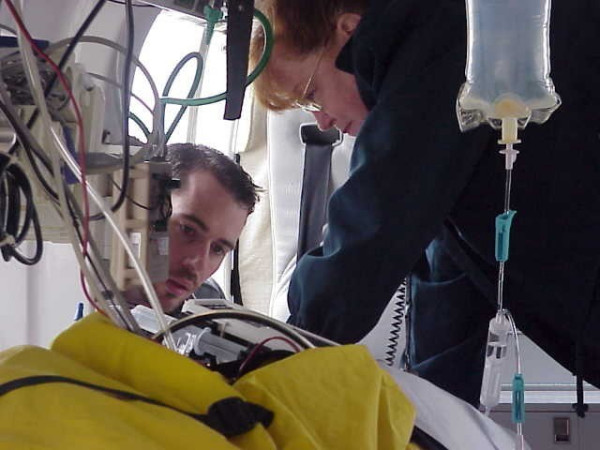
Instructors Making Finals Preparation for Simulation.

A Laerdal SimMan™ was loaded onto a BK117 EMS equipped helicopter and situated in the same way as a live patient (Figures 1, 2, 3, 4). The simulator was connected to shore power through an extension cord inserted through the door of the helicopter. The simulator is capable of powering itself through a compressed air tank and a battery pack; however, the cost of actual flight was prohibitive, therefore, shore power was used. The monitors and laptop computer required to operate the simulator used battery power.

The aircraft was run at flight idle setting to provide noise, vibration, lighting, olfactory input and heat similar to the conditions encountered during flight. The largest vibration acceleration magnitudes on the airframe tend to occur at the main rotor passage frequency. Typical 4-blade helicopters have rotor passage frequencies between 17 and 20 Hz. The vibration is multi-directional, with similar magnitudes in all three translational axes. The highest vibration magnitudes occur during landing, take-off or maneuvering, all of which involve hovering and low speed flight[25]. During cruise, combined ride values[26] of around 0.8 ms^-2 ^r.m.s. are expected, which are likely to be "fairly uncomfortable" for seated personnel. Measurements in transport helicopters suggest that vibration magnitudes experienced while standing on the ground are typical of those that occur in cruising flight, provided that the engines are running at flight idle; in aircraft where it is normal practice to reduce the engine revolutions while standing on the ground, the rotation frequencies will be lowered, altering the spectral shape of the vibration[25].

An experienced operator managed the simulator while two of the investigators functioned as flight nurse and observer. Each study subject was given a one minute briefing concerning the simulated patient's medical condition, prior to boarding the helicopter. One of two pre-programmed simulations was conducted for each resident. The first simulation mimicked a young woman who had been involved in a motor vehicle accident. The simulation began after she had been loaded into the helicopter on a backboard with cervical spine protection in place and with one intravenous line established. Shortly after the simulation starting point, the patient exhibited signs of hypovolemia and tension pnuemothorax and requires fluid resuscitation, needle thoracostomy, and endotracheal intubation. The second simulation represented a middle-aged man sustaining an anterior myocardial infarction. He had received thrombolytic therapy and was being transferred to a tertiary care hospital for acute intervention. At the beginning of the simulation he underwent ventricular fibrillation and required advanced cardiac life support and endotracheal intubation.

### Measurements

**Figure 5:**
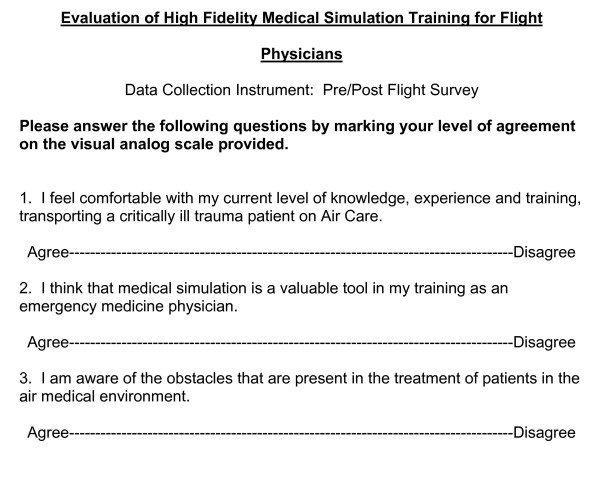
Pre- Post-Simulation Questionnaire.

Prior to participating in the simulation, the resident physicians completed a brief questionnaire (Figure 5). The questionnaire included visual analog scales measuring the residents' self-efficacy. We defined self-efficacy as the self-perceived comfort in their ability to care for the patient in the helicopter environment, the value of the simulation for training, and recognition of obstacles to treatment. After each simulated flight the resident completed the same questionnaire, and a semi-structured interview was conducted to gather qualitative data (Table 1). This interview was taped and subsequently transcribed for interpretation and analysis.

Over the next three months, all twelve residents, both the simulation and the non-simulation groups, completed a questionnaire after their first non-simulated flight as a flight physician. This questionnaire surveyed prior pre-hospital experience and quantified self-perceived comfort, self-perceived ability to care for the patient in the helicopter, and pre-flight awareness obstacles in the helicopter setting. The simulation group was also questioned about the perceived value of the simulation. The questions are shown as the headings for Table 2, and use a five point Likert scale.

### Data analysis

Continuous data were compared between pre- and post-simulation using a paired samples t-test. Categorical data were compared using Fisher's Exact test. The audiotapes from the semi-structured interviews were transcribed verbatim into electronic format. The transcriptions were evaluated by two investigators for common themes and areas for expanding the simulation for training in prehospital care. Data were analyzed using SPSS v 13.0 (SPSS Inc., Chicago, Il)

## Results

### Participants

The simulation and non-simulation groups were similar in their prehospital experiences; one resident in each group had experience in the air medical transport of critically ill patients prior to residency. Demographics were similar; mean age in the simulation group was 29 years, and 6 were male while mean age in the non-simulation group was 30 years, all 5 were male.

### Feasibility

The simulation event was carried out successfully. All participants completed one simulation each. Two of the simulations were interrupted by software difficulties that arose secondary to the use of a unfamiliar computer to operate the SimMan™ operating system. Despite this difficulty, all simulations were run to completion after a software restart. The costs of the simulation were $440 and 22 total hours of highly skilled instructor and technician time. (Table 3)

### Self-efficacy

Compared to before the training, participating residents tended to be more comfortable and more aware of obstacles to treating patients in the air medical environment after their training was completed (Figure 1), although this was only statistically significant for awareness of obstacles (p = 0.029). There were no significant differences in self-assessed comfort level, ability to use the equipment, or knowledge of obstacles between those who undertook training and those who did not. Every resident undertaking the simulation agreed it was educational, it should be included in residency training, and that it improved their comfort (Table 2).

Common themes in the structured interview that were agreed upon by the two transcript reviewers included recognition of the value of the training, particularly of the realistic environment. Most felt more prepared to fly in the future and some noted the need to prepare more fully prior to their first flight. Most felt that simulation, though not perfect, was a useful addition to their training and should be incorporated as a regular part of residency training.

## Discussion

High fidelity medical simulation provides a patient-safe means of educating medical trainees, and is becoming common at multiple levels of medical education. We have demonstrated that it is feasible to carry the simulation experience into the challenging environment of an air ambulance helicopter and provide a high quality experience. While we did experience several problems with simulation implementation, these would have been avoided through increased experience with the Laerdal SimMan and further knowledge of the computer used to control the simulator. Even with these problems, this experience was deemed as valuable by the learners and actively demonstrated to them the limitations and challenges of the flight medicine environment. Simulation provides the opportunity to improve self-confidence in a patient safe context. This information may be generalized to other challenging arenas ranging from the battlefield to the trauma room. Medical simulation no longer needs to be relegated only to a discrete education laboratory environment. It can be brought to the practice arena and allow learners to acquire the skills, knowledge and attitudes required to effectively practice in a specific environment. Simulation may provide an important opportunity for training in critical care and lifesaving procedures in all manors of challenging settings. Further study of the benefits of high fidelity simulation in austere environments and in the teaching of rarely performed procedures is warranted.

While our experience was feasible and self-assessed as efficacious by the participants, it was expensive and labor intensive for our relatively small educational endeavor. Table 3 lists the cost break down by dollar amount and time spent. This table does not include the amortized costs of the simulator nor the helicopter. Nor does it account for the preparation time which included writing simulation programs for SimMan on the included software package, design of the exercise, establishment of goals and objectives and coordination of the event. All high quality educational events are labor intensive and require extensive planning; but this event, like most high fidelity simulation exercises was inordinately resource intensive and had the added expense of the helicopter and pilot.

## Limitations

The limitations of this study include small sample size and selection bias through self-selection of groups. However the primary purpose of this study was to prove feasibility of simulation in the challenging environment. Secondarily, we attempted to measure self-efficacy and cost. All conclusions concerning material learned were gathered for hypothesis generation only.

A second area of limitation arose from the helicopter running at flight idle. While the conditions in the helicopter running at flight idle on the ground are typical of those encountered during transport, the conditions encountered during take off, landing, and maneuvering are not simulated. Although the SimMan is capable of running from compressed air and therefore being useable in the air, the cost of seven flights would have been several thousand dollars and would be prohibitive for a pilot feasibility program. If further work demonstrates that the high fidelity simulation improves performance, the cost of training may be easier to justify.

## Conclusion

We concluded that high fidelity simulation is feasible, but expensive to implement in the challenging environment of the air medical setting. We recommend that those wishing to implement this technology become familiar with operating the simulator *in situ *prior to commencing training. We found high fidelity simulation to improve self-assessed recognition of the challenges present in the prehospital, flight environment, and that Emergency Medicine residents value high fidelity simulation as an aspect of their training in this resource-limited, physically challenging arena.

## Competing interests

The author(s) declare that they have no competing interests.

## Authors' contributions

Each author made the following contributions to the manuscript:

SW contributed to the study concept and design, acquisition of the data, analysis and interpretation of the data, drafting of the manuscript, critical revision of the manuscript for important intellectual content, statistical expertise, obtained funding, administrative, technical, or material support, study supervision

CL contributed to the study concept and design, analysis and interpretation of the data, critical revision of the manuscript for important intellectual content, and statistical expertise

WH contributed to the acquisition of the data and critical revision of the manuscript for important intellectual content

AW contributed to the acquisition of the data and critical revision of the manuscript for important intellectual content

CH contributed to the study concept and design, acquisition of the data and critical revision of the manuscript for important intellectual content

CHL provided critical revision of the manuscript for important intellectual content

GH contributed to the critical revision of the manuscript for important intellectual content and provided administrative, technical, or material support

All authors read and approved the final manuscript.

## Pre-publication history

The pre-publication history for this paper can be accessed here:



## Figures and Tables

**Figure 6:**
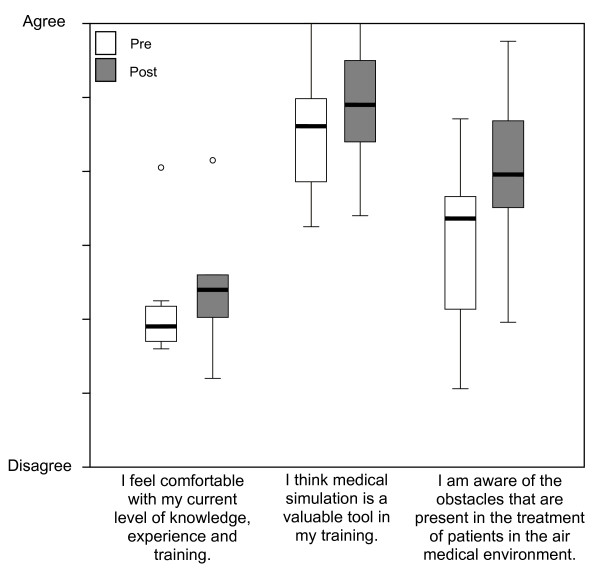
Level of agreement with statements offered pre- and post-training (results of VAS questionnaire).

**Table 1:** Semi-Structured Interview Questions

Was this exercise a valuable use of your time?
What were the strengths of this exercise?
What were the weaknesses of this exercise?
What could be done to further improve this exercise?
Do you feel more or less prepared fro your upcoming first flight as the Air Care Flight Physician? Why or why not?
What did you learn about Air-Medical Medicine today?
What did you learn about medical simulation today?
Should this experience become a regular part of residency training?

**Table 2:** Responses to the survey for each resident, stratified by whether or not they undertook the training

		**During my first three flights I...**		**Prior to my first flight...**	**The human patient simulator...**		
		...felt comfortable with my level of knowledge, experience and training	...was able to utilize all of the equipment and supplies necessary for patient care while working in the aircraft	...I was aware of the obstacles present in the treatment of patients in the air medical environment	...was educational	...should be included in the residency training program	...helped me feel more comfortable on my first patient care experience as a flight physician
**No training**	Subject 1	+	+	+			
	Subject 2	+	o	+			
	Subject 3	+	+	+			
	Subject 4^$^	+	+	+			
	Subject 5	-	-	o			

**Training**	Subject 6	+	+	-	+	+	+
	Subject 7	-	+	o	+	+	+
	Subject 8^$^	o	+	+	+	+	+
	Subject 9	o	-	o	+	+	+
	Subject 10	+	o	+	+	+	+
	Subject 11	o	o	+	+	+	+
	Subject 12	o	o	o	+	+	+

P-value		0.086	0.773	0.735			

+ Agree, - Disagree, o Neither agree nor disagree

^$ ^Residents who had prior experience of transporting critically ill patients

**Table 3:** Estimated Cost of Simulation

**COST**	**COMMENT**	**TOTAL**
Simulator Operator	Set Up – 2 hours Simulation – 3 hours Tear Down – 2 hours	7 hours
Onboard Instructors	2 physicians functioning as educators and flight personnel (3 hours each)	6 hours
Offboard Instructors	2 physicians Pre- and Post-Debrief (3 hours each)	6 hours
Pilot	3 hours	$200.00
Helicopter	3 hours of flight idle settings 20 gal/hr Jet Air-A $4.00/gal	$240.00

TOTAL	22 hours	$440.00
